# Biliverdin incorporation into the cyanobacteriochrome SPI1085g3 from *Spirulina*

**DOI:** 10.3389/fmicb.2022.952678

**Published:** 2022-08-02

**Authors:** Xian-Jun Wu, Jia-Ying Qu, Chang-Tian Wang, Ya-Ping Zhang, Ping-Ping Li

**Affiliations:** ^1^College of Biology and the Environment, Nanjing Forestry University, Nanjing, China; ^2^Collaborative Innovation Center of Sustainable Forestry in Southern China of Jiangsu Province, Nanjing Forestry University, Nanjing, China; ^3^National Positioning Observation Station of Hung-tse Lake Wetland Ecosystem in Jiangsu Province, Hongze, China

**Keywords:** cyanobacteriochrome, biliverdin, photoswitchable fluorescent protein, dark reversion, *Spirulina*

## Abstract

Cyanobacteriochromes (CBCRs) bind linear tetrapyrrole chromophores, mostly phycocyanobilin (PCB), and exhibit considerable spectral diversity with a high potential for biotechnological applications. Particular attention has been given to the conversion into intrinsic biliverdin (BV) incorporation due to the absence of PCB in mammalian cells. Our recent study discovered that a red/green CBCR of *Spirulina subsalsa*, SPI1085g3, was covalently attached to PCB and exhibited strong red fluorescence with a unique red/dark switch. In this study, we found that SPI1085g3 could be modestly chromophorylated with BV and absorb somewhat shifted (10 nm) red light, while the single C448S mutant could efficiently bind BV and exhibit unidirectional photoconversion and moderate dark reversion. The fluorescence in its dark-adapted state was switched off by red light, followed by a moderate recovery in the dark, and these were properties similar to those of PCB-binding SPI1085g3. Furthermore, by introducing the CY motif into the conserved CH motif for chromophore attachment, we developed another variant, C448S_CY, which showed increased BV-binding efficiency. As expected, C448S_CY had a significant enhancement in fluorescence quantum yield, reaching that of PCB-binding SPI1085g3 (0.14). These BV-binding CBCRs offer an improved platform for the development of unique photoswitchable fluorescent proteins compared with PCB-binding CBCRs.

## Introduction

Cyanobacteriochromes (CBCRs) are photoswitchable linear, tetrapyrrole-binding photoreceptor proteins that are found to exist only in cyanobacteria (Ikeuchi and Ishizuka, 2008). They require only a GAF (cGMP-specific phosphodiesterase/adenylate cyclase/FhlA) domain for the incorporation of a linear tetrapyrrole serving as a sensory module, among which red/green CBCRs that utilize phycocyanobilin (PCB) to detect red light in most cases have been deeply studied and characterized (Fushimi and Narikawa, 2019). The typical red/green CBCRs bind to a PCB chromophore *via* a conserved canonical Cys residue within their GAF domains and show reversible photoconversion between the red-absorbing dark-adapted state and the green-absorbing photoproduct state, which is triggered by Z/E isomerization of a double bond between the C15 and C16 positions of the chromophore (Narikawa et al., 2008; Zhang et al., 2010; Rockwell et al., 2012, 2015, 2022; Pennacchietti et al., 2015; Wiebeler et al., 2019; Xu et al., 2020; Kirpich et al., 2021; Rao et al., 2021; Song et al., 2021). These domains are considered excellent candidates for bioimaging applications due to their small molecular size, natural monomeric state, remarkable spectral diversity, and especially interesting photoswitching behavior. However, the PCB chromophore that binds to abundant CBCRs is mainly present in cyanobacteria but absent in mammalian cells. Although exogenous addition and endogenous synthesis of PCB in cells provide alternative approaches, there are complex procedures (Müller et al., 2013; Beyer et al., 2015; Uda et al., 2017, 2020). Moreover, some naturally occurring far-red-sensing CBCRs (frCBCRs) derived from the chlorophyll (Chl) *d*-containing marine cyanobacteria *Acaryochloris marina* and *Leptolyngbya* sp. bind not only PCB but also biliverdin (BV, Figure 6A; Narikawa et al., 2015a,b; Fushimi et al., 2016; Blain-Hartung et al., 2018; Moreno et al., 2020), which is abundant in many potential host cells, including mammalian tissues, and represents a longer wavelength-absorbing chromophore to facilitate deeper penetration into the tissues (Chen et al., 2021; Manoilov et al., 2022). Therefore, more attention has been given to PCB replacement with BV to incorporate CBCRs for advantages in applications (Fushimi et al., 2019, 2020; Oliinyk et al., 2019; Buhrke, 2021; Tachibana et al., 2021; Tang et al., 2021; Ma et al., 2022).

*Spirulina* is a freshwater cyanobacterium in the alkaline water of volcanic lakes and is phylogenetically distinct from *Acaryochloris marina* and *Leptolyngbya* sp. that harvest far-red light (Moreno et al., 2020). We have recently reported two novel PCB-binding CBCR GAF domains, SPI1085g2 and SPI1085g3, isolated from *Spirulina subsalsa* (Wu et al., 2018; Jiang et al., 2021). PCB-binding SPI1085g2 exhibited typically reversible photoconversion between the green light-absorbing photoproduct and the red light-absorbing dark-adapted state but only with weak fluorescence (Jiang et al., 2021), while PCB-binding SPI1085g3 exhibited unidirectional photoconversion and moderate dark reversion from the orange-absorbing photoproduct to the red-absorbing dark-adapted state with intense fluorescence (Wu et al., 2018). The fluorescence could be switched off by illumination with red light, followed by a moderate recovery in the dark. Therefore, SPI1085g3-PCB exhibits a unique red/dark switch and offers a platform for the development of unique photoswitchable fluorescent proteins.

In this study, we tried to replace PCB with BV for incorporation into SPI1085g3 and found that wild-type SPI1085g3 modestly attached BV, while the single C448S mutant could efficiently bind BV with a chromophore-binding efficiency comparable with that of the previously reported AM1_1557g2 (Narikawa et al., 2015a). BV-binding C448S exhibited red fluorescence with a unique red/dark switch, which is similar to that of PCB-binding SPI1085g3 but represents a better platform for the development of unique photoswitchable fluorescent proteins due to BV incorporation. A further mutant, C448S_CY, bound BV more efficiently than C448S with a high fluorescence quantum yield, which provided an advantage for bioimaging applications.

## Materials and methods

### Plasmid construction

For PCB-binding protein expression, the His-tagged SPI1085g3 (amino acid positions 397–551) (Supplementary Figure 4) and fused ho1:pcyA (encoding enzymes for PCB synthesis) genes were inserted into two multiple clone sites of a pETDuet-1 vector (Novagen, Madison, WI, United States) in *E. coli* (Wu et al., 2018). For BV-binding protein expression, the SPI1085g3 and ho1 (encoding the enzyme for BV synthesis) genes were also inserted into two multiple clone sites of a pETDuet vector (Wu et al., 2018). For the replacement of Cys448 of SPI1085g3 with Ser, site-directed mutagenesis was performed with appropriate primers using the aforementioned two constructs as templates and the Fast Site-Directed Mutagenesis Kit (Tiangen Biotech, Beijing, China). The primer sets of the forward primer (5′-TGTGGTGGGGAAAAACTCCCCAATTATTTCG-3′) and reverse primer (5′-GAGTTTTTCCC CACCACAGTCCGCCATTCTT-3′) were prepared. Furthermore, the replacement of His484 of SPI1085g3 with Tyr was performed using the C448S construct as the template with the appropriate nucleotide primer set (5′-TACCTCCAAATGCTAGAACAATTACAAGCCC-3′, 5′-ACACGGGCTA AAACCCACCTCATAAATATCCG-3′). The primers were synthesized by GenScript. The sequences of all constructs were verified by nucleotide sequencing.

### Protein expression and purification

The aforementioned plasmids were separately transformed into *E. coli* BL21 (DE3). The transformed cells were cultured in 100 ml of Luria-Bertani (LB) medium supplemented with ampicillin (20 μg/ml) at 37°C until the optical density at 600 nm was 0.4–0.8, and then the cells were kept in an ice bath for 30 min. Subsequently, isopropyl β-D-thiogalactoside (IPTG) was added to the culture media at a final concentration of 1 mM, and protein expression was induced overnight at 18°C. The cells were harvested by centrifugation at 12,000 × *g* for 5 min at 4°C and suspended in 20 mM potassium phosphate buffer (KPB), pH 7.0, containing 0.5 M NaCl. All proteins were extracted and purified as described in a previous study (Wu et al., 2018).

### Protein analysis and modeling

The purified proteins were boiled and denatured with 2 × SDS (2% (w/v) sodium dodecyl sulfate) sample buffer containing 30 mM β-mercaptoethanol for 5 min and then subjected to SDS–PAGE [10% (w/v) acrylamide]. The electrophoresed gels were soaked in 1.5 M zinc acetate at room temperature for 30 min, and the bilins in the samples were detected by Zn^2+^-induced fluorescence. Fluorescence was visualized through a 630-nm filter upon excitation at 530 nm (GenoSens1850, Clinx, Shanghai, China). The gels were further stained with Coomassie brilliant blue, and the purified proteins were visualized.

Homology modeling of the three-dimensional structure was performed on the Swiss Model Server using the reported crystal structure of miRFP670nano (PDB ID code: 6MGH) (Oliinyk et al., 2019). The chromophore BV (Figure 6A) was taken from miRFP670nano and directly docked to the chromophore pocket of the modeling structure using PyMOL.^1^ The figures were created using PyMOL.

### Spectral analysis

All experiments were performed at room temperature. Absorption spectra of the proteins were recorded using a PerkinElmer Lambda 365 spectrophotometer. BV-binding efficiency was approximately estimated based on the absorption maximum of the biliproteins in the visible region and protein absorption at 280 nm (A_max_/A_280_) (Narikawa et al., 2015a). A cold fiberoptic light source with a 150 W halogen lamp (Bocheng, Nanjing, China) was used to generate monochromic red light through a bandpass filter (Rayan, Changchun, China) to induce photoconversion as previously described (Wu et al., 2018). Light intensity at the sample plane for photoconversion was 15 μmol/m^2^/s^1^. To monitor the dark reversion of the biliproteins, the absorbance at a wavelength of 652 nm (or 642 nm for the PCB-binding protein) after red light irradiation was recorded at intervals of 5 s for 30 min under dark conditions. Scanning kinetics during dark reversion for C448S_CY were recorded at intervals of 2 min, and the absorbance at a wavelength of 638 nm was monitored at intervals of 1 min to determine the dark reversion kinetics. The half-lives were calculated from the kinetics of dark reversion by exponential fitting. BV-binding proteins were denatured using 8 M urea at pH 2.0 for 15 min in the dark. Likewise, the denatured proteins were irradiated with white light for 3 min. The absorption spectra were recorded before and immediately after irradiation.

Fluorescence emission spectra were measured on a model LS 55 spectrofluorimeter (PerkinElmer, Waltham, MA, United States) with an excitation of 600 nm for the Pr states of biliproteins and 560 nm for the Po states. Fluorescence excitation spectra were monitored by emission wavelength at 710 nm for the Pr states and 670 nm for the Po states. The slits were set at 10 nm for both excitation and emission, and the scan speed was 1,200 nm/min. Fluorescence quantum yields, Φ_F_, were determined in KPB (pH 7.0) using the known Φ_F_ = 0.27 of C-PC from *Nostoc* (Zhang et al., 2010) as the standard. To estimate fluorescence recovery kinetics during dark reversion, the fluorescence intensities at 680 nm (or 662 nm for the PCB-binding protein) were measured at 1-min intervals for 30 min. The spectra were obtained with an integration time of 0.1 s, and the exciting light was turned off automatically during intervals. Fluorescence scanning kinetics for C448S_CY were recorded at intervals of 5 min, and the fluorescence intensity at a wavelength of 672 nm was monitored at intervals of 1 min to determine the fluorescence recovery kinetics. The half-lives were calculated from the kinetics of fluorescence recovery by exponential fitting.

### Microscopic analysis

The cells containing a plasmid expressing biliprotein were induced and grown for 12 h. After harvesting, the cells were washed with distilled water and deposited on a glass slide. Micrographs of the live cells were recorded with a fluorescence microscope (Axio Vert. A1, Carl Zeiss Microscopy GmbH, Jena, Germany) equipped with a cooled CCD camera (Axiocam 503 color, Carl Zeiss, Jena, Germany). The CCD camera was controlled by ZEN 2 imaging software. Upon excitation at 600/25 nm, fluorescence images were obtained through a 685/25-nm filter. The bright-field images of the same areas were further captured under white light illumination.

## Results

### Chromophorylation of SPI1085g3 with biliverdin

**FIGURE 1:**
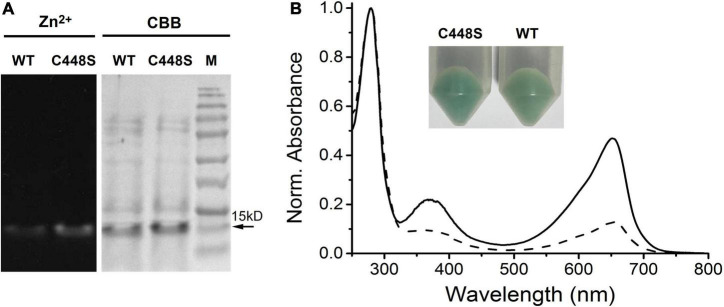
Chromophorylation of SPI1085g3 and C448S with BV. **(A)** SDS–PAGE analyses of SPI1085g3 and C448S chromophorylated with BV: zinc-induced fluorescence (Zn^2+^, left) and Coomassie brilliant blue (CBB, right) staining. The marker (SMOBiO, PM2510) indicates protein molecular weights of 180, 140, 100, 75, 60, 45, 35, 25, 15, and 10 kDa from top to bottom. **(B)** Absorbance spectra of BV-incorporated SPI1085g3 (dashed line) and C448S (solid line). Protein absorbance values at 280 nm were adjusted to be equal. The color of *E. coli* cell pellets expressing the wild-type SPI1085g3 (WT) and C448S variant (C448S) is shown in the inset.

We previously reported a unique red/dark switchable CBCR, SPI1085g3, bound to PCB from *Spirulina* (Wu et al., 2018). To utilize BV instead of PCB as a chromophore, SPI1085g3 was coexpressed in *E. coli* with heme oxygenase for BV production. Preliminarily, SPI1085g3 could be chromophorylated with BV, as determined by the deep blue–green color in *E. coli* cell pellets (Figure 1B, inset). Next, purified SPI1085g3 isolated from *E. coli* cells was subjected to SDS–PAGE. There was one protein band at approximately 15 kDa based on Coomassie brilliant blue staining (Figure 1A right), which is consistent with the calculated molecular mass of His-tagged SPI1085g3. Only a small amount of BV was bound to SPI1085g3, as demonstrated by a band with weak zinc-induced fluorescence observed by SDS–PAGE (Figure 1A left). Furthermore, BV-binding SPI1085g3 exhibited an absorption spectrum with a maximum peak at 652 nm and a shoulder at approximately 600 nm (Figure 1B dashed line). The BV-binding efficiency of SPI1085g3 was low (12.79 ± 2.1%) (Table 1 and Figure 1B dashed line) compared with that of the reported far-red-sensing AM1_1557g2 (Narikawa et al., 2015a), AM1_C0023g2 (Fushimi et al., 2016), and AM1_6305g2 (Fushimi et al., 2019) from *Acaryochloris marina* and close to that of AM1_1870g3 (Narikawa et al., 2015b) and AM1_1186g2 (Kuwasaki et al., 2019) but significantly higher than that of the red sensing AnPixJg2, AnPixJg4, and NpF2164g5 from chlorophyll *d*-lacking cyanobacteria (Fushimi et al., 2019). Furthermore, irradiation of SPI1085g3 with red light resulted in a decrease in red light absorption (Pr) and an increase in the orange light region (Po) (Supplementary Figure 2A). The absorption maxima of the denatured SPI1085g3 Pr and Po states were observed at ∼676 and ∼612 nm, respectively (Supplementary Figure 2B). Moreover, fluorescence excitation and emission spectra of the SPI1085g3 Pr state were obtained (Supplementary Figure 2C heavy lines), with peak emission at 680 nm and a quantum yield of only 0.01 (Table 1); fluorescence excitation and emission spectra of the SPI1085g3 Po state were obtained (Supplementary Figure 2C thin lines), with peak emission at 663 nm and a fairly weak fluorescence. In this context, we considered that SPI1085g3 can be moderately chromophorylated with BV.

**TABLE 1:** Biochemical properties of BV-binding SPI1085g3 (WT) and its variants.

Biliprotein	BV-binding efficiency	Absorbance				Fluorescence			Half-life
			
		λ_max_ [nm]		ε [M^–1^⋅cm^–1^] × 10^5^		λ_max_ [nm]		Φ_F_	*t*_1/2_ [min]
					
		15Z	15E	15Z	15E	15Z	15E	15Z	15E to 15Z
WT	13 ± 2.1%	652	590	0.88 ± 0.05	0.24 ± 0.02	680	663	0.01	–
C448S	50 ± 2.3%	652	578	0.86 ± 0.06	0.21 ± 0.03	680	660	0.04	3.2
C448S_CY	65% ± 7.3%	638	573	0.84 ± 0.01	0.24 ± 0.02	672	–	0.14	40.5

### Efficient biliverdin incorporation

Our previous report showed that the replacement of Cys448 with Ser, C448S, improved the PCB-binding efficiency of SPI1085g3 (Wu et al., 2018). In this context, we speculated that a similar improvement could also possibly occur for BV incorporation. As expected, the *E. coli* cell pellets expressing C448S showed a deeper blue–green color than those expressing the wild-type protein (Figure 1B inset), suggesting that the binding efficiency and/or expression level of C448S were higher than those of the wild-type protein. Next, the purified C448S was subjected to SDS-PAGE. There was one band at approximately 15 kDa corresponding to C448S, which was consistent with that of the wild-type protein (Figure 1A left). C448S showed strong zinc-dependent fluorescence based on a small amount of C448S protein compared with the wild-type protein (Figure 1A right), suggesting that the C448S mutant had a higher BV-binding efficiency than the wild-type protein. Furthermore, the BV-binding C448S mutant exhibited a red-absorbing (Pr) state with an absorbance peak at 652 nm (Table 1 and Figure 1B solid line), which was identical to that of the wild-type protein. Unexpectedly, the BV-binding efficiency of C448S was 50 ± 2.3% (Table 1 and Figure 1B solid line), which was approximately fourfold higher than that of the wild-type protein and comparable with that of most previously reported BV-binding CBCRs (Narikawa et al., 2015a,b; Fushimi et al., 2019; Kuwasaki et al., 2019).

### Photoconversion and dark reversion

**FIGURE 2:**
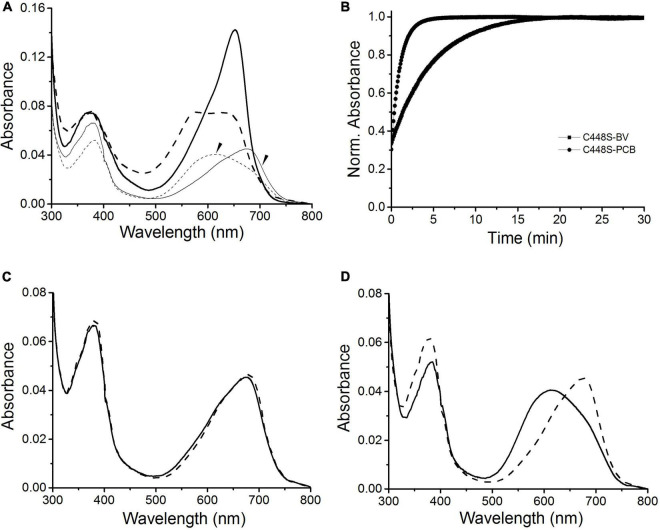
Photoconversion and dark reversion of BV-binding C448S. **(A)** Absorbance spectra of native (heavy lines) and acid-urea denatured (thin lines indicated by the arrow) biliprotein. The dashed lines correspond to the native (heavy dashed line) and denatured (thin dashed line indicated by the arrow) 15E state obtained after irradiation with 653/20 nm light; **(B)** absorbance at 652 nm monitored after driving the biliprotein (C448S-BV) to the E-state. C448S-PCB represents the PCB-binding C448S as a control. **(C)** Absorbance spectra of the acid-denatured SPI1085g3 Po state: absorbance spectra just after denaturation (solid line) and after light illumination (dashed line). **(D)** Absorbance spectra of the acid-denatured SPI1085g3 Pr state: absorbance spectra just after denaturation (solid line) and after light illumination (dashed line).

Because of the high efficiency of C448S for BV incorporation, we further characterized the photochemical properties of C448S. When photoconversion was performed by intense illumination with 653 nm light, C448S exhibited an orange-absorbing (Po) photoproduct state with an absorbance maximum at approximately 578 nm (Table 1 and Figure 2A), accompanied by a decrease in red absorption. Immediately, reversion from the Po state to the Pr state was observed in the dark when illumination was stopped (Figure 2A heavy lines). We measured the dark reversion kinetics of BV-binding C448S at a peak wavelength of 652 nm. The protein exhibited a moderate dark reversion with a half-life of 3.2 min (Table 1 and Figure 2B), which was significantly slower than that of PCB-binding C448S, with a half-life of 15.3 s (Wu et al., 2018). The result was opposite to those of AM1_1557g2, AM1_1870g3, and AM1_C0023g2 (Narikawa et al., 2015a,b; Fushimi et al., 2016), where dark reversions of the BV-binding proteins were much faster than the PCB-binding ones. To identify the chromophore species and their configurations, spectra were obtained for acid-denatured C448S. Absorption maxima of the denatured C448S Pr and Po states were observed at ∼676 and ∼612 nm, respectively (Figure 2A thin lines indicated by the arrow). Irradiation of the denatured Po state with orange light resulted in a redshift of the absorption spectra (Figure 2C), whereas irradiation of denatured Pr with red light resulted in no appreciable spectral change (Figure 2D). These results indicate *15Z* and *15E* configurations for the chromophore in the Pr and Po states, respectively.

### Fluorescent properties

**FIGURE 3:**
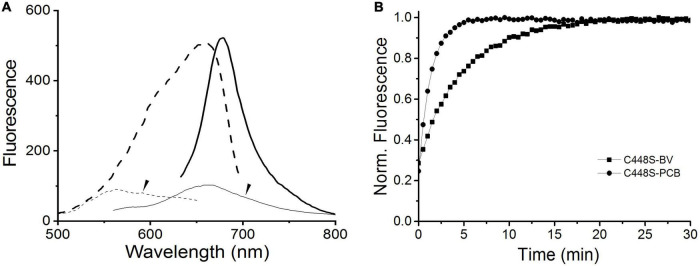
Fluorescence properties of BV-binding C448S. **(A)** Fluorescence excitation (dashed lines) and emission (solid lines) spectra of the C448S Pr state (heavy lines) and the C448S Po state (thin lines indicated by the arrow). **(B)** Normalized fluorescence intensity of the biliprotein (C448S-BV) at 680 nm during dark reversion. C448S-PCB represents the PCB-binding C448S as a control. The half-lives calculated by exponential fitting are 3.2 min for C448S-BV and 15.3 s for C448S-PCB.

To evaluate the potential of BV, instead of PCB, bound to C448S as a fluorescent imaging probe, we measured the fluorescence excitation and emission spectra of the C448S Pr state at room temperature. This BV-binding Pr state fluoresced with a maximum value at 680 nm and exhibited excitation spectra peaking at 652 nm (Figure 3A heavy lines), corresponding to its absorption spectra, which were redshifted compared with those of the PCB-binding one with an emission peak at 662 nm and an excitation peak at 642 nm (Wu et al., 2018). The fluorescence quantum yield of BV-binding C448S was calculated to be 4% and was much lower than that of PCB-binding C448S (15%) (Wu et al., 2018). The C448S Po state exhibited a weak fluorescence with an emission peak at 660 nm (Figure 3A dashed lines). Furthermore, the fluorescence of the BV-binding Pr state could be switched off by red light irradiation, which was followed by photoconversion and then by a moderate fast recovery in the dark. The kinetics of fluorescence recovery was determined based on the increase in fluorescence at 680 nm (Figure 3B). The rate of fluorescence recovery, which corresponded to its dark reversion rates of absorbance, was much lower than that of PCB-binding C448S and reverted to that of the wild-type SPI1085g3 bound to PCB, which can be used as a unique red/dark switch (Wu et al., 2018). The half-life obtained from the fluorescence kinetics was 3.2 min, which was identical to that obtained from the corresponding absorbance kinetics.

### Improvement in fluorescence quantum yield

**FIGURE 4:**
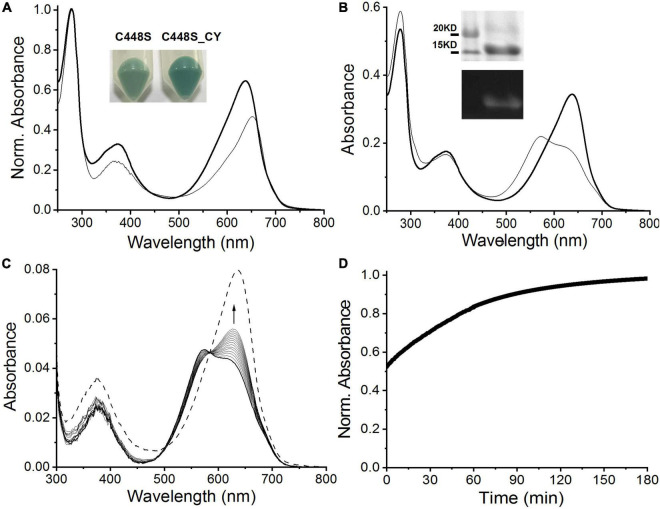
Characterization of C448S_CY. **(A)** Absorbance spectra of C448S_CY (heavy solid line) and C448S (thin solid line) as a control. Protein absorbance values at 280 nm were adjusted to be equal. In the inset, the color of *E. coli* cell pellets is shown for expressed C448S_CY (right) and C448S (left) as a control. **(B)** Photoconversion of C448S_CY. The thin solid line corresponds to the 15E state obtained after irradiation with 653/20 nm light. In the inset, the total protein (up) and covalently bound bilin (down) are shown for purified C448S_CY. **(C)** Absorbance spectra of C448S_CY acquired at the indicated times after irradiation. The dashed line indicates absorbance spectra of the 15Z state obtained before irradiation. **(D)** Normalized absorbance at 638 nm monitored after driving the biliprotein to the E-state.

To improve the fluorescence quantum yield of C448S, we reviewed the comparison of the primary sequence of SPI1085g3 with those of other red/green CBCRs (Wu et al., 2018) and focused on a highly conserved CH motif required for chromophore attachment (Supplementary Figure 1), as nearly all red/green CBCRs, except NpF2164g5, which contained a CY motif and showed high fluorescence quantum yield (Rockwell et al., 2012; Fushimi et al., 2019), contained the CH motif. Thus, we replaced the His residue with the Tyr residue in the C448S mutant. The new mutant, C448S_CY, showed an obvious improvement in BV incorporation with a 65 ± 7.3% binding efficiency based on absorption spectra (Table 1 and Figure 4A). The cell pellet expressing C448S_CY also showed a deeper green color than that expressing C448S (Figure 4A, inset). However, C448S_CY exhibited a blueshifted Pr state with an absorbance peak at 638 compared with that of C448S (Table 1 and Figure 4A). The C448S_CY protein was subjected to SDS-PAGE. The Coomassie blue-stained SDS-PAGE gel showed a band at approximately 15 kDa that emitted fluorescence in the presence of zinc ions (Figure 4B, inset), indicating the binding of BV to the protein. Furthermore, C448S_CY exhibited photoconversion from the Pr state to the Po state under red light irradiation, which was also blueshifted with an absorbance maximum at 573 (Figure 4B). Moreover, C448S_CY was subjected to acid denaturation, and the absorption maxima of the denatured Pr and Po states were observed at ∼676 and ∼612 nm, respectively, which were consistent with those of C448S (Supplementary Figure 3), indicating the common chromophore species and configuration arrangement. Subsequently, we measured the dark reversion of the C448S_CY Po state. Significantly slow dark reversion from the Po to Pr state was observed by absorbance spectra acquired at the indicated times (2 min) after red irradiation (Figure 4C). The dark reversion half-life was 40.5 min, as determined by detecting the increase in absorbance at 638 nm (Figure 4D). There was an approximately 12-fold increase compared with that of C448S (Table 1).

**FIGURE 5:**
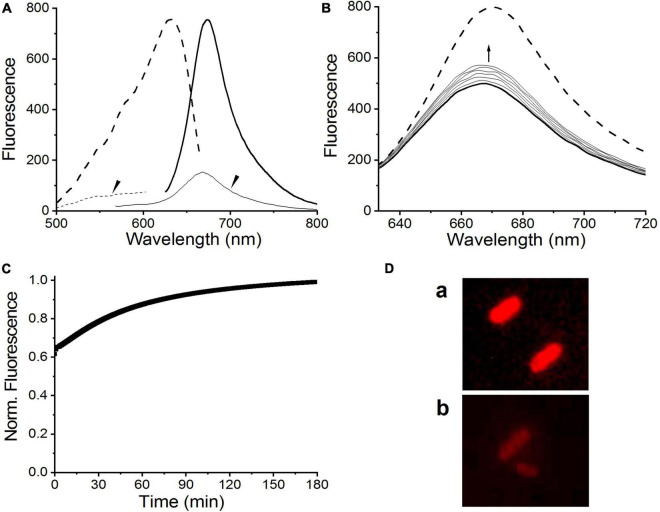
Fluorescence properties of C448S_CY. **(A)** Fluorescence excitation (dashed lines) and fluorescence emission spectra (solid lines) of the C448S_CY Pr state (heavy lines) and the C448S_CY Po state (thin solid lines indicated by the arrow). **(B)** Fluorescence spectra of C448S_CY acquired at the indicated times during dark reversion; and **(C)** fluorescence intensity at 672 nm during dark reversion. The dashed line indicates fluorescence emission spectra of the 15Z state obtained before irradiation. **(D)** Fluorescence images of *E. coli* cells expressing C448S_CY **(a)** and C448S **(b)**.

Fluorescence excitation and emission spectra were measured to evaluate the potential of C448S_CY for applications in bioimaging. As expected, BV-binding C448S_CY exhibited strong red fluorescence with an emission maximum at 672 nm (Figure 5A heavy solid line), which showed a blueshift of 8 nm compared with that of BV-binding C448S and resulted in a redshift of only 10 nm compared with the fluorescence emission peak at 662 nm of PCB-binding C448S (Wu et al., 2018). Furthermore, unexpectedly, the fluorescence quantum yield of C448S_CY was calculated as 0.14 (Table 1), which was sevenfold higher than that of C448S and reached that of PCB-binding SPI1085g3. Furthermore, the fluorescence of the C448S_CY Po state was not detected using an appropriate excitation wavelength (Figure 5A, thin lines indicated by the arrow). To further evaluate the imaging potential of the BV-binding proteins, the micrographic fluorescence properties in live cells were examined. *E. coli* cells expressing C448S_CY showed stable red fluorescence (Figure 5D), which was much brighter than that of C448S due to the high fluorescence quantum yield (Figure 5D up and down). The Pr fluorescence of C448S_CY could be switched off and then recovered slowly (Figure 5B). The kinetics of fluorescence recovery by monitoring the increase in fluorescence at 672 nm was consistent with the increase in absorbance during dark reversion (Figure 5C). The half-life obtained from the fluorescence kinetics was 40.8 min, which was basically consistent with that obtained from the corresponding absorbance kinetics.

**FIGURE 6:**
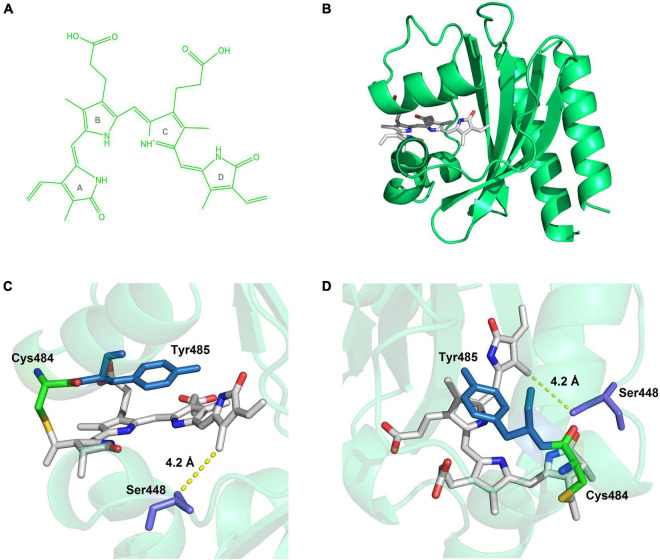
Simulated structure of C448S_CY. **(A)** The structure of biliverdin. **(B)** The overall view of the BV-incorporated C448S_CY. **(C)** The side view close to the pocket, Ser448 surrounds the D ring of the chromophore, at the closest distance of 4.2 Å. **(D)** The above view, the benzene ring of Tyr485 is parallel to the C-ring.

## Discussion

In this study, we found that SPI1085g3, a reported typical red/green CBCR from *Spirulina subsalsa*, could moderately bind BV and exhibit a red-absorbing dark-adapted state rather than a far-red-absorbing dark-adapted state, unlike chlorophyll *d*-bearing cyanobacteria. These far-red-sensing CBCRs incorporate BV derived from the cyanobacterium *Acaryochloris marina* (Narikawa et al., 2015a,b; Fushimi et al., 2016, 2019; Kuwasaki et al., 2019) and *Leptolyngbya* sp. strain JSC-1 (Gan et al., 2014; Blain-Hartung et al., 2018; Moreno et al., 2020), which specifically synthesizes chlorophyll *d* to capture far-red light as an energy source. This effect could be considered a result of the coevolution of photosynthesis and light perception to adapt to the far-red light-enriched environment. For photosynthesis, most classical cyanobacteria, such as *Spirulina* in this study, can only synthesize chlorophyll *a* and lack chlorophyll *d*. Therefore, their CBCRs are almost barely attached to BV. This finding may imply that cyanobacteria utilized CBCRs bound to BV not only to detect far-red light but also to detect red light, which was only somewhat redshifted compared with that of the CBCRs bound to PCB, suggesting a unique strategy of *Spirulina subsalsa*’s adaptation to a red-light environment. Furthermore, some BV-binding CBCRs from *Nostoc flagelliforme* (Xu et al., 2022), a well-known desert cyanobacterium, were found to suggest using BV to sense light environments may be a widespread mechanism in cyanobacteria.

Protein engineering based on rational mutagenesis is an effective and attractive approach to CBCR improvement for bioimaging tools (Fushimi et al., 2019; Kuwasaki et al., 2019). The C448S mutation in SPI1085g3 exhibited significant improvement for linear tetrapyrrole incorporation, not just for PCB incorporation but more importantly for BV incorporation. This result implied that two chromophores covalently attached to SPI1085g3 may exhibit a similar binding mode for favorable chromophore accommodation. Because of the C3^1^ binding of the PCB chromophore in reported red/green CBCRs based on structural information (Narikawa et al., 2013; Rockwell et al., 2015; Fushimi et al., 2019; Xu et al., 2020), a similar situation might also be expected in the cases of the PCB and BV chromophores in SPI1085g3. Furthermore, slower dark reversion of the BV-binding C448S than that of the PCB-binding C448S was opposite to those found for AM1_1557g2, AM1_1870g3, and AM1_C0023g2 (Narikawa et al., 2015a,b; Fushimi et al., 2016), where fast and relatively slow dark reversions of BV and PCB-binding C448S were observed. This implied that C448S differed from these reported CBCRs in the structural arrangement of the chromophore-binding pockets, in which the BV chromophore was considered the C3^2^-binding mode. Notably, the Cys/Ser position is not conserved in extensive red/green CBCRs based on multiple sequence alignments (Wu et al., 2018). The Cys/Ser residue is equivalent to Ile285 in AnPixJg2_BV4 (Supplementary Figure 1), which are residues positioned toward, but distant from, ring D of the chromophores (Narikawa et al., 2013; Fushimi et al., 2019). Val54 of miRFP670nano corresponding to Cys/Ser448 does not directly interact with BV based on its crystal structure (Supplementary Figure 1; Oliinyk et al., 2019). Furthermore, we built a homology model for C448S_CY based on the structure of miRFP670nano (Figure 6B) and found that Ser448 surrounds the D ring of BV within 6 Å of the chromophore (Figure 6C). Therefore, this replacement of Cys with Ser may indirectly affect the local environment surrounding the D ring to facilitate chromophore incorporation.

C448S bound to BV showed a small spectral redshift (5∼15 nm) in comparison with that of C448S bound to PCB. This bilin-dependent shift was considerably less pronounced for most reported red/green CBCRs, whether natural or artificial. In other words, C448S bound to BV exhibited significantly blueshifted absorption and fluorescence spectra compared with those of maximum CBCRs bound to BV. Similar spectral blueshifts were observed for an engineered fluorescent variant of NpR3784, miRFP670nano (Oliinyk et al., 2019), which exhibited C3^1^ attachment of BV with a double bond between C3^1^ and C3^2^. Therefore, C448S probably possesses a similar C3^1^ binding mode related to the blueshifted spectra. The blueshifted property offers an opportunity for the development of biosensors based on FRET and multicolor bioimaging in red and far-red optical windows (Hontani et al., 2016; Shcherbakova et al., 2016; Baloban et al., 2017; Oliinyk et al., 2019).

Notably, the C448S mutant showed a sharp decrease in fluorescence quantum yield due to the replacement of PCB with BV. However, by introducing the CY motif into the conserved CH motif for chromophore attachment in C448S, the fluorescence quantum yield of BV-binding C448S_CY was dramatically improved and recovered to that of PCB-binding SPI1085g3 (Wu et al., 2018) and exceeded that of most BV-binding CBCRs, such as bright fluorescent miRFP670nano (Oliinyk et al., 2019, 2022), required by 17 rounds of directed molecular evolution and bearing 18 residue substitutions. The critical substitution, H87Y, corresponded to CH replacement with CY, indicating that the CY motif has a remarkable effect on the fluorescence quantum yield of chromophore-binding CBCRs. From the homology model for C448S_CY, the benzene ring of the Tyr residue of the CY motif is exactly parallel to the C-ring on the chromophore (Figure 6D). They formed a perfect π–π stacking of each other. The interaction of this conjugate system was likely to favor the stabilization of the chromophore and affect the spectral properties of the chromophore, causing shifts in absorption and fluorescence spectra. We noted that C448S_CY exhibited a blueshift of approximately 14 nm in the absorption spectrum compared with that of C448S, which was consistent with the above assumption. Furthermore, this resulted in a 7 nm blueshift in peak fluorescence emission and a significant increase in fluorescence quantum yield. In addition, C448S_CY showed a larger Stokes shift (34 nm) than C448S and even most biliproteins, including phycobiliproteins (Wu et al., 2013; Rodriguez et al., 2016) and phytochromes (Fischer and Lagarias, 2004; Oliinyk et al., 2017). The Stokes shifts of most biliproteins usually do not exceed 25 nm. This is considered one of the major disadvantages of bilin-binding fluorescent proteins. The CH motif of wild red/green CBCRs except NpR2164g5 bearing the CY motif is highly conserved based on multiple sequence alignments (Wu et al., 2018). Therefore, NpR2164g5 with a high fluorescence quantum yield and C448S_CY may share a common molecular mechanism. Relying on the CY motif, the chromophore-binding pocket of these CBCRs stabilized not only the dark-adapted state but also the photoproduct, which was consistent with the slow dark reversion of the C448S_CY Po state. Furthermore, photoconversion of miRFP670nano and NpR2164g5 (Rockwell et al., 2012) could not be detected, implying a solid dark-adapted state and an extremely low quantum yield for photoconversion. They could be classified into a group of red-inactive CBCRs (Rockwell et al., 2022). Due to the CY motif, C448S_CY exhibited an increased BV-binding efficiency, a relatively large Stokes shift, and significantly enhanced fluorescence. The fluorescence of the biliproteins with photoconversion, including PCB-binding SPI1085g3 (Wu et al., 2018), can be turned off. There seems to be a limitation in imaging applications because the excitation light may trigger photoconversion, leading to a non-fluorescent state. The solution is to use the appropriate excitation light that does not cause a large photoconversion. Photoconversion is triggered by a much stronger light than the excitation light. The fluorescence imaging could be performed with the weaker light for excitation and, if necessary, the stronger light for repression. When the irradiation for repression is stopped, the fluorescent molecules with moderate dark reversion could be automatically lighted up. In principle, this might provide a unique optical regulation approach for bioimaging and analysis of protein dynamics.

To date, a large number of CBCRs bound to PCB have been found and characterized with remarkable spectral diversity. The replacement of PCB with BV to bind to CBCRs is advantageous and attractive for future applications in bioimaging, even without considering the advantage of the spectral redshift that reduces autofluorescence; the reason is that the BV chromophore is present in a wider range of organisms, including mammals, whereas PCB is restricted to cyanobacteria, red algae, and cryptomonads. Moreover, even though linear tetrapyrrole chromophores are produced by heterologous expression of bilin synthases in cells, the synthesis of BV, a precursor of the reduced PCB in the biosynthetic pathway, is simpler than that of PCB because no ferredoxin-dependent bilin reductases are needed. Therefore, an attempt to convert the numerous PCB-binding CBCRs into BV-acceptable molecules is of great value. Although some engineered BV-binding phycobiliproteins have higher fluorescence quantum yields than CBCRs bound to BV (Rodriguez et al., 2016; Hou et al., 2019), they are not photochromic. Furthermore, the low chromophore-binding efficiency and fluorescence quantum yield should be improved for practical use based on molecular evolution approaches. Therefore, these BV-binding CBCRs with unique spectral characteristics are expected to be an excellent platform for the development of bioimaging tools.

## Data availability statement

The original contributions presented in the study are included in the article/Supplementary material, further inquiries can be directed to the corresponding author.

## Author contributions

X-JW conceived and designed the experiments. X-JW, J-YQ, C-TW, and Y-PZ performed the experiments. X-JW and J-YQ analyzed the data. X-JW and P-PL contributed reagents, materials, and analysis tools and wrote the manuscript. All authors contributed to the article and approved the submitted version.

## Footnotes

1
http://pymol.org
