# Supplementary material for: Biliverdin incorporation into the cyanobacteriochrome SPI1085g3 from Spirulina
Source: Front Microbiol. 2022 Aug 2;13:952678. doi: 10.3389/fmicb.2022.952678 (PMC9378818; doi:10.3389/fmicb.2022.952678)
Supplement: Supplementary file 1 [file Data_Sheet_1.PDF]

Supplementary Material

Supplementary Figures

```
SPI1085g3       (416-464) ERVVIYHFLPDWSGEFMA--ESKGEEWRAVVGKNC-PILSDRHLRETQGGRY
miRFP670nano      (22-67) DRVCVFQFEEDYSGVVVV--EAVDDRWISILKTQV----RDRYFMETRGEEY
NpF2164g5_BV4   (889-938) DRLAVFRFHPDWSVEFVA--ESVKDKWLSLADSDIKTIWMDEYLQETQGGRY
AnPixJg2_BV4    (253-302) DRVAVYRFNPDWSGEFVA--ESVGSGWVKLVGPDIKTVWEDTYLQETQGGRY
AM1_1870g3_BV4  (531-581) DHVAVYQFDENWGGSFINNFRAVKPEWEEVVYSTR-DVWNDSYLQETKGGRY
AM1_1557g2      (236-285) DRVVVYKFFPDWSGEFLV--EATAPNILPLSELEVPMVWQDTYLQENQGGKY
AM1_C0023g2     (270-319) DRVVIYQFWPDWSGEFLV--ESTAPGLIPLSELDVPMTWQDTYLQENQGGKF

SPI1085g3       (465-516) AAHETSMVTDIYEAGFSPCHLQMLEQLQARAYMIVPIFLGENLWGLLAAYQN
miRFP670nano     (68-119) SHGRYQAIADIYTANLTECYRDLLTQFQVRAILAVPILQGKKLWGLLVAHQL
NpF2164g5_BV4   (938-990) RNHETTVVNDIYTVGYVQCYLEILEKIQAKAYAVAPIFIGNKLWGFIGAYQN
AnPixJg2_BV4    (303-354) RHQESTVVNDIYEAGYFSCHLEILEQFEIKAYIVVPVFAAEKLWGLLAAYQN
AM1_1870g3_BV4  (582-633) RHNHVTVVNDVSKAGYSPCHLETYHYYQIKAFLVAPVFVGSRLWGLIGAYQH
AM1_1557g2      (286-337) RDNATTVVADIYQESYRDCHLEILEWYKIRAYMVVPVFIGETLWGLLAAYQL
AM1_C0023g2     (320-371) KDNAPTVVADIYQQSYTDCHLEILEWFDIRAYMVVPVFIGKTLWGLLAAYQL
```

Supplementary Figure 1 Sequence alignment of SPI1085g3 and some reported BV-binding CBCR GAF domains. Highly conserved residues in CBCR GAF domains, black in bold; residues conserved in red/green CBCR GAF domains, red; BV4 residues, orange; C448 position, green; the highly conserved CH motif for chromophore attachment, blue.

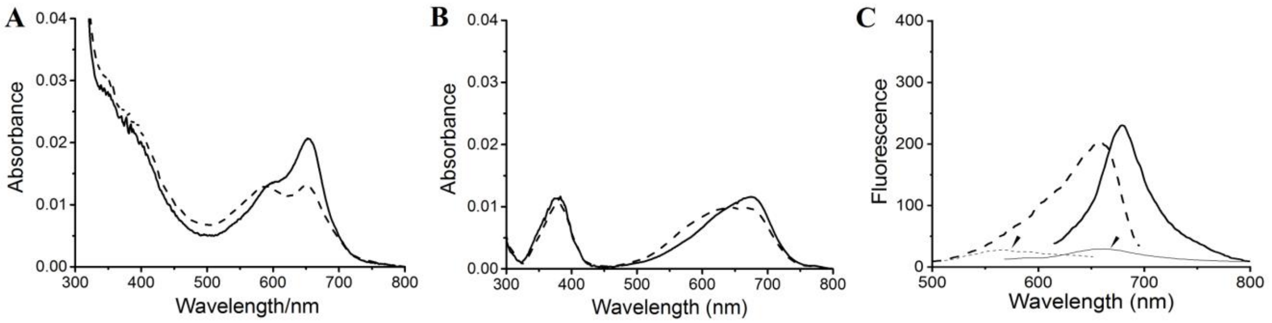

Supplementary Figure 2 Spectral properties of wild-type SPI1085g3 (WT) bound to BV. Absorbance spectra of native (A) and acid-urea denatured (B) WT. The dashed lines correspond to the 15E state obtained after irradiation with 653/20 nm light. (C) Fluorescence excitation (dashed lines) and emission (solid lines) spectra of the WT Pr state (heavy lines) and the WT Po state (thin lines indicated by the arrow).

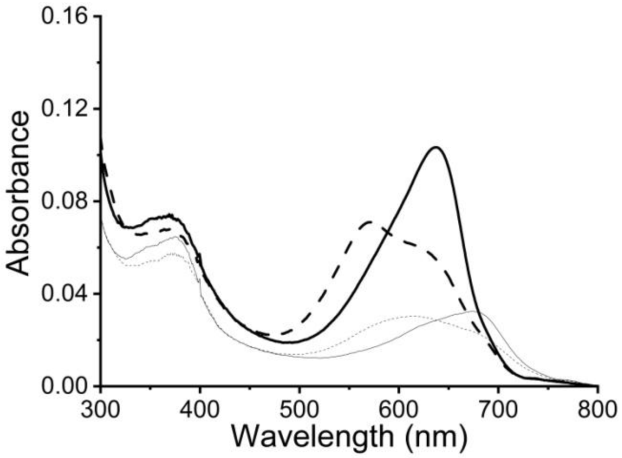

Supplementary Figure 3 Absorbance spectra of native (heavy lines) and acid-urea denatured (thin lines) C448S bound to BV. The dashed lines correspond to the 15E state obtained after irradiation with 653/20 nm light.

His-tag::SPI1085g3

MGSSHHHHHHSQDPNSSSIRRSLNLQTIFNTATQEVRQVLQAERVVIYRFLPDWSGEFMAESKGEEWRTVVGKNCPI

ISDKHLRETQGGRYAAHETSTVTDIYEVGFSPCHLQMLEQLQARAYMIVPIFLGENLWGLLAAYQNSAPRYWQADEV

ELLTQIGSQLGMAIQQGQYLQQMQAQS

His-tag::C448S

MGSSHHHHHHSQDPNSSSIRRSLNLQTIFNTATQEVRQVLQAERVVIYRFLPDWSGEFMAESKGEEWRTVVGKNSPI

ISDKHLRETQGGRYAAHETSTVTDIYEVGFSPCHLQMLEQLQARAYMIVPIFLGENLWGLLAAYQNSAPRYWQADEV

ELLTQIGSQLGMAIQQGQYLQQMQAQS

His-tag::C448S_CY

MGSSHHHHHHSQDPNSSSIRRSLNLQTIFNTATQEVRQVLQAERVVIYRFLPDWSGEFMAESKGEEWRTVVGKNSPI

ISDKHLRETQGGRYAAHETSTVTDIYEVGFSPCYLQMLEQLQARAYMIVPIFLGENLWGLLAAYQNSAPRYWQADEV

ELLTQIGSQLGMAIQQGQYLQQMQAQS

Supplementary Figure 4 Amino acid sequences of the expressed constructs. Single underlines and double underlines denote the fragments of the original genes of CBCRs， and the His-tags, respectively.
